# 1,1′-(*p*-Phenyl­enedimethyl­ene)dipyridinium bis­(hexa­fluoridophosphate)

**DOI:** 10.1107/S1600536810037992

**Published:** 2010-09-30

**Authors:** Munirah Sufiyah Abdul Rahim, Yatimah Alias, Seik Weng Ng

**Affiliations:** a Department of Chemistry, University of Malaya, 50603 Kuala Lumpur, Malaysia

## Abstract

The title salt, C_18_H_18_N_2_^2+^·2PF_6_^−^, exists as non-inter­acting cations and anions. In the cation, the pyridine and phenyl­ene rings are aligned at 62.9 (1)°; the pyridine ring lies on a special position of *m* site symmetry and the phenyl­ene ring on a special position of 2/*m* site symmetry. The angle at the methyl­ene C atom is 112.8 (1)°. The anion lies on a special position of *m* site symmetry; four F atoms lie on this mirror plane.

## Related literature

For the tetra­phenyl­borate salt, see: Wu *et al.* (2007 ▶) and for the tetra­cyano­quinodimethanide salt, see: Ashwell *et al.* (1975 ▶); Hudson & Robson (2009 ▶).
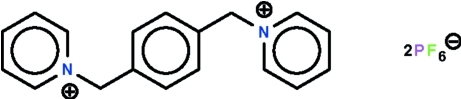


## Experimental

### 

#### Crystal data

C_18_H_18_N_2_^2+^·2PF_6_^−^
*M* *_r_* = 552.28
Orthorhombic, 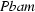

*a* = 11.1013 (11) Å
*b* = 12.6742 (12) Å
*c* = 7.3483 (7) Å
*V* = 1033.91 (17) Å^3^
*Z* = 2
Mo *K*α radiation
μ = 0.33 mm^−1^
*T* = 100 K
0.30 × 0.20 × 0.10 mm

#### Data collection

Bruker SMART APEX diffractometer
Absorption correction: multi-scan (*SADABS*; Sheldrick, 1996 ▶) *T*_min_ = 0.908, *T*_max_ = 0.968
6200 measured reflections
1280 independent reflections
1121 reflections with *I* > 2σ(*I*)
*R*_int_ = 0.028

#### Refinement

*R*[*F* ^2^ > 2σ(*F* ^2^)] = 0.029
*wR*(*F* ^2^) = 0.088
*S* = 1.05
1280 reflections
91 parameters
H-atom parameters constrained
Δρ_max_ = 0.33 e Å^−3^
Δρ_min_ = −0.44 e Å^−3^

### 

Data collection: *APEX2* (Bruker, 2009 ▶); cell refinement: *SAINT* (Bruker, 2009 ▶); data reduction: *SAINT*; program(s) used to solve structure: *SHELXS97* (Sheldrick, 2008 ▶); program(s) used to refine structure: *SHELXL97* (Sheldrick, 2008 ▶); molecular graphics: *X-SEED* (Barbour, 2001 ▶); software used to prepare material for publication: *publCIF* (Westrip, 2010 ▶).

## Supplementary Material

Crystal structure: contains datablocks global, I. DOI: 10.1107/S1600536810037992/jh2207sup1.cif

Structure factors: contains datablocks I. DOI: 10.1107/S1600536810037992/jh2207Isup2.hkl

Additional supplementary materials:  crystallographic information; 3D view; checkCIF report

## Figures and Tables

**Fig. 1.**
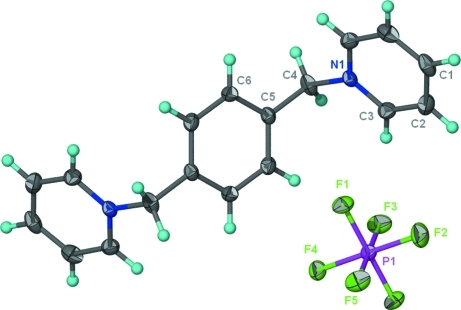
Thermal ellipsoid plot (Barbour, 2001) of C18H18N22+ 2PF6- at the 70% probability level; hydrogen atoms are drawn as spheres of arbitrary radius.

## supplementary crystallographic information

### Comment

The structure of the 1,1'-(4-dimethylphenylene)dipyridinium cation has been reported in a number of examples (Ashwell *et al.*, 1975; Hudson & Robson, 2009; Wu *et al.*,2007). We ourselves have reported other examples. The title hexafluorophosphate (Scheme I, Fig. 1) exists as non-interacting cations and anions. In the cation, the pyridyl and phenylene rings are aligned at 62.9 (1) °. The angle at the methylene C atom is 112.8 (1) °. The anion lies on a mirror plane such that four F atoms lie within the mirror plane.

### Experimental

α,α'-Dibromo-*p*-xylene (5.28 g, 20 mmol) was dissolved in acetonitrile (30 ml) and to the solution was added pyridine (2.96 g, 40 mmol). The solution was heated for 2 h. The solid product was recrystallized from a methanol/ethanol mixture to afford 1,1'-(4-dimethylphenylene)dipyridinium bromide. The bromide ion was exchanged by the hexafluorophosphate ion by reaction of the salt (1 mmol) with ammonium hexafluorophosphate (2 mmol) in water. The reactants were mixed in water for 2 h to give a solid material. This was collected and recrystallized from acetonitrile.

### Refinement

Carbon-bound H-atoms were placed in calculated positions (C—H 0.95 to 0.99 Å) and were included in the refinement in the riding model approximation, with *U*(H) set to 1.2*U*(C).

### Crystal data

**Table:**

C_18_H_18_N_2_^2+^·2PF_6_^−^	*F*(000) = 556
*M**_r_* = 552.28	*D*_x_ = 1.774 Mg m^−^^3^
Orthorhombic, *P**b**a**m*	Mo *K*α radiation, λ = 0.71073 Å
Hall symbol: -P 2 2ab	Cell parameters from 2836 reflections
*a* = 11.1013 (11) Å	θ = 2.8–28.2°
*b* = 12.6742 (12) Å	µ = 0.33 mm^−^^1^
*c* = 7.3483 (7) Å	*T* = 100 K
*V* = 1033.91 (17) Å^3^	Block, colorless
*Z* = 2	0.30 × 0.20 × 0.10 mm

### Data collection

**Table:**

Bruker SMART APEX diffractometer	1280 independent reflections
Radiation source: fine-focus sealed tube	1121 reflections with *I* > 2σ(*I*)
graphite	*R*_int_ = 0.028
ω scans	θ_max_ = 27.5°, θ_min_ = 2.4°
Absorption correction: multi-scan (*SADABS*; Sheldrick, 1996)	*h* = −14→14
*T*_min_ = 0.908, *T*_max_ = 0.968	*k* = −12→16
6200 measured reflections	*l* = −9→8

### Refinement

**Table:**

Refinement on *F*^2^	Primary atom site location: structure-invariant direct methods
Least-squares matrix: full	Secondary atom site location: difference Fourier map
*R*[*F*^2^ > 2σ(*F*^2^)] = 0.029	Hydrogen site location: inferred from neighbouring sites
*wR*(*F*^2^) = 0.088	H-atom parameters constrained
*S* = 1.05	*w* = 1/[σ^2^(*F*_o_^2^) + (0.0505*P*)^2^ + 0.3897*P*] where *P* = (*F*_o_^2^ + 2*F*_c_^2^)/3
1280 reflections	(Δ/σ)_max_ < 0.001
91 parameters	Δρ_max_ = 0.33 e Å^−^^3^
0 restraints	Δρ_min_ = −0.44 e Å^−^^3^

### Fractional atomic coordinates and isotropic or equivalent isotropic displacement parameters (Å^2^)

**Table:**

	*x*	*y*	*z*	*U*_iso_*/*U*_eq_	Occ. (<1)
P1	0.25173 (4)	0.14923 (4)	0.0000	0.01624 (16)	
F1	0.25218 (7)	0.14917 (6)	0.21872 (11)	0.0238 (2)	
F2	0.31281 (13)	0.26387 (9)	0.0000	0.0351 (3)	
F3	0.12080 (10)	0.20151 (10)	0.0000	0.0281 (3)	
F4	0.19348 (10)	0.03444 (8)	0.0000	0.0238 (3)	
F5	0.38395 (10)	0.09687 (10)	0.0000	0.0282 (3)	
N1	0.00180 (13)	0.29642 (11)	0.5000	0.0151 (3)	
C1	0.17001 (16)	0.45395 (14)	0.5000	0.0227 (4)	
H1	0.2269	0.5100	0.5000	0.027*	
C2	0.12837 (12)	0.41294 (11)	0.3374 (2)	0.0231 (3)	
H2	0.1579	0.4391	0.2247	0.028*	
C3	0.04351 (11)	0.33362 (10)	0.34067 (18)	0.0192 (3)	
H3	0.0143	0.3050	0.2296	0.023*	
C4	−0.09367 (16)	0.21325 (14)	0.5000	0.0203 (4)	
H4A	−0.1451	0.2225	0.3911	0.024*	0.50
H4B	−0.1451	0.2225	0.6089	0.024*	0.50
C5	−0.04278 (15)	0.10309 (13)	0.5000	0.0153 (4)	
C6	−0.02152 (11)	0.05152 (10)	0.66385 (17)	0.0181 (3)	
H6	−0.0364	0.0867	0.7758	0.022*	

### Atomic displacement parameters (Å^2^)

**Table:**

	*U*^11^	*U*^22^	*U*^33^	*U*^12^	*U*^13^	*U*^23^
P1	0.0184 (3)	0.0162 (3)	0.0141 (3)	−0.00093 (16)	0.000	0.000
F1	0.0274 (4)	0.0300 (5)	0.0140 (4)	−0.0014 (3)	−0.0013 (3)	−0.0022 (3)
F2	0.0502 (8)	0.0217 (6)	0.0333 (7)	−0.0156 (6)	0.000	0.000
F3	0.0272 (6)	0.0346 (7)	0.0224 (6)	0.0125 (5)	0.000	0.000
F4	0.0309 (6)	0.0196 (5)	0.0210 (6)	−0.0068 (5)	0.000	0.000
F5	0.0189 (6)	0.0400 (7)	0.0255 (6)	0.0042 (5)	0.000	0.000
N1	0.0150 (6)	0.0115 (6)	0.0187 (7)	0.0018 (5)	0.000	0.000
C1	0.0148 (8)	0.0134 (8)	0.0398 (11)	0.0014 (6)	0.000	0.000
C2	0.0222 (6)	0.0212 (6)	0.0260 (7)	0.0022 (5)	0.0048 (5)	0.0064 (5)
C3	0.0220 (6)	0.0197 (6)	0.0161 (6)	0.0030 (5)	−0.0002 (5)	0.0003 (5)
C4	0.0148 (8)	0.0136 (8)	0.0323 (10)	−0.0005 (6)	0.000	0.000
C5	0.0129 (7)	0.0130 (8)	0.0201 (9)	−0.0020 (6)	0.000	0.000
C6	0.0215 (6)	0.0167 (6)	0.0159 (6)	−0.0027 (5)	0.0021 (5)	−0.0021 (5)

### Geometric parameters (Å, °)

**Table:**

P1—F4	1.5921 (11)	C1—H1	0.9500
P1—F3	1.5975 (12)	C2—C3	1.3779 (18)
P1—F2	1.6034 (12)	C2—H2	0.9500
P1—F1^i^	1.6072 (8)	C3—H3	0.9500
P1—F1	1.6072 (8)	C4—C5	1.506 (2)
P1—F5	1.6107 (12)	C4—H4A	0.9900
N1—C3^ii^	1.3444 (15)	C4—H4B	0.9900
N1—C3	1.3444 (15)	C5—C6	1.3902 (15)
N1—C4	1.495 (2)	C5—C6^ii^	1.3902 (15)
C1—C2	1.3828 (18)	C6—C6^iii^	1.391 (2)
C1—C2^ii^	1.3828 (18)	C6—H6	0.9500
			
F4—P1—F3	90.54 (7)	C2^ii^—C1—H1	120.2
F4—P1—F2	178.95 (7)	C3—C2—C1	119.18 (14)
F3—P1—F2	90.51 (7)	C3—C2—H2	120.4
F4—P1—F1^i^	90.05 (3)	C1—C2—H2	120.4
F3—P1—F1^i^	90.17 (3)	N1—C3—C2	120.45 (13)
F2—P1—F1^i^	89.95 (3)	N1—C3—H3	119.8
F4—P1—F1	90.05 (3)	C2—C3—H3	119.8
F3—P1—F1	90.17 (3)	N1—C4—C5	112.81 (14)
F2—P1—F1	89.95 (3)	N1—C4—H4A	109.0
F1^i^—P1—F1	179.64 (7)	C5—C4—H4A	109.0
F4—P1—F5	89.64 (7)	N1—C4—H4B	109.0
F3—P1—F5	179.82 (7)	C5—C4—H4B	109.0
F2—P1—F5	89.31 (7)	H4A—C4—H4B	107.8
F1^i^—P1—F5	89.83 (3)	C6—C5—C6^ii^	120.02 (16)
F1—P1—F5	89.83 (3)	C6—C5—C4	119.97 (8)
C3^ii^—N1—C3	121.11 (16)	C6^ii^—C5—C4	119.97 (8)
C3^ii^—N1—C4	119.44 (8)	C5—C6—C6^iii^	119.99 (8)
C3—N1—C4	119.44 (8)	C5—C6—H6	120.0
C2—C1—C2^ii^	119.60 (17)	C6^iii^—C6—H6	120.0
C2—C1—H1	120.2		
			
C2^ii^—C1—C2—C3	1.7 (3)	C3—N1—C4—C5	90.39 (12)
C3^ii^—N1—C3—C2	−1.6 (2)	N1—C4—C5—C6	91.26 (13)
C4—N1—C3—C2	177.67 (13)	N1—C4—C5—C6^ii^	−91.26 (13)
C1—C2—C3—N1	−0.1 (2)	C6^ii^—C5—C6—C6^iii^	0.3 (3)
C3^ii^—N1—C4—C5	−90.39 (12)	C4—C5—C6—C6^iii^	177.77 (16)

Symmetry codes: (i) *x*, *y*, −*z*; (ii) *x*, *y*, −*z*+1; (iii) −*x*, −*y*, *z*.
